# Epidemiology of *Plasmodium* infections in Flores Island, Indonesia using real-time PCR

**DOI:** 10.1186/1475-2875-12-169

**Published:** 2013-05-24

**Authors:** Maria MM Kaisar, Taniawati Supali, Aprilianto E Wiria, Firdaus Hamid, Linda J Wammes, Erliyani Sartono, Adrian JF Luty, Eric AT Brienen, Maria Yazdanbakhsh, Lisette van Lieshout, Jaco J Verweij

**Affiliations:** 1 Department of Parasitology, Faculty of Medicine, University of Indonesia, Jakarta, Indonesia; 2 Department of Parasitology, Center of Infectious Diseases, Leiden University Medical Center, Albinusdreef 2, Leiden, 2333 ZA, The Netherlands; 3 Department of Microbiology, Faculty of Medicine, University of Hasanuddin, Makassar, Indonesia; 4 Unités Mixtes de Recherche, Institut de Recherche pour le Développement, Mère et Enfant Face aux Infections, Tropicales, France; 5 Current address: Laboratory for Medical Microbiology and Immunology, St. Elisabeth Hospital, Tilburg, The Netherlands

**Keywords:** Malaria, *Plasmodium falciparum*, *Plasmodium vivax*, *Plasmodium malariae*, Real-time PCR, Diagnosis, Flores

## Abstract

**Background:**

DNA-based diagnostic methods have been shown to be highly sensitive and specific for the detection of malaria. An 18S-rRNA-based, real-time polymerase chain reaction (PCR) was used to determine the prevalence and intensity of *Plasmodium* infections on Flores Island, Indonesia.

**Methods:**

Microscopy and real-time multiplex PCR for the detection of *Plasmodium* species was performed on blood samples collected in a population-based study in Nangapanda Flores Island, Indonesia.

**Results:**

A total 1,509 blood samples were analysed. Real-time PCR revealed prevalence for *Plasmodium falciparum*, *Plasmodium vivax*, and *Plasmodium malariae* to be 14.5%, 13.2%, and 1.9% respectively. Sub-microscopic parasitaemia were found in more than 80% of all positive cases. The prevalence of *P. falciparum* and *P. vivax* was significantly higher in subjects younger than 20 years (p ≤ 0.01). In the present study, among non-symptomatic healthy individuals, anaemia was strongly correlated with the prevalence and load of *P. falciparum* infections (p ≤ 0.01; p = 0.02) and with the load of *P. vivax* infections (p = 0.01) as detected with real-time PCR. Subjects with AB blood group tend to have a higher risk of being infected with *P. falciparum* and *P. vivax* when compared to other blood groups.

**Conclusion:**

The present study has shown that real-time PCR provides more insight in the epidemiology of *Plasmodium* infections and can be used as a monitoring tool in the battle against malaria. The unsurpassed sensitivity of real-time PCR reveals that sub microscopic infections are common in this area, which are likely to play an important role in transmission and control.

**Trial registration:**

Trials number ISRCTN83830814.

## Background

In Indonesia, 1.25 to 2.5 million probable malaria cases with 45 to 50% being cases of *Plasmodium falciparum*, nearly 350,000 confirmed malaria cases, and around 500 confirmed malaria deaths, are reported every year [1]. Based on the Annual Parasite Incidence data the highest risk of acquiring malaria is in the eastern part of Indonesia. In 2008, a mass blood survey in more than 200,000 subjects performed by the Basic Health Research Institute of East Nusa Tenggara showed 15% of the blood slides to be positive for malaria parasites [2].

Although microscopic diagnosis of malaria is easy to perform, consumable costs are low, and it allows quantification and species identification [3,4], in many malaria-endemic areas microscopy of thin and thick smears of peripheral blood remains challenging. It is time consuming and needs well-trained and dedicated personnel, and even then misdiagnosis in cases of low parasitaemia and incorrect species identification occurs frequently [5-7].

Alternatively, DNA-based detection methods have been developed targeting different *Plasmodium* genes showing high sensitivity and specificity. The 18S ribosomal RNA gene has been used successfully as a target for malaria diagnosis in conventional polymerase chain reaction (PCR) and real-time PCR in several studies [8-11]. These assays allow sensitive far below the detection limit of microscopy and specific detection and differentiation of the different *Plasmodium* species [12-14]. Moreover, real-time PCR is not only able to provide information on parasite species but it also provides quantitative data on the parasite load.

In the present study, a multiplex real-time PCR was used to determine the epidemiological profile of *Plasmodium* infections in a malaria-endemic area in Flores, Indonesia.

## Methods

This study was approved by the ethical committee from Faculty of Medicine, University of Indonesia in ref: 194/PT02.FK/ETIK/2006 and has been filed by ethical committee of the Leiden University Medical Centre. The present cross-sectional observational study is part of a larger study on co-infection between malaria and soil-transmitted helminths in Ende district [15], NTT which is listed in SPIN-KNAW projects [16].

### Study area

**Figure 1:**
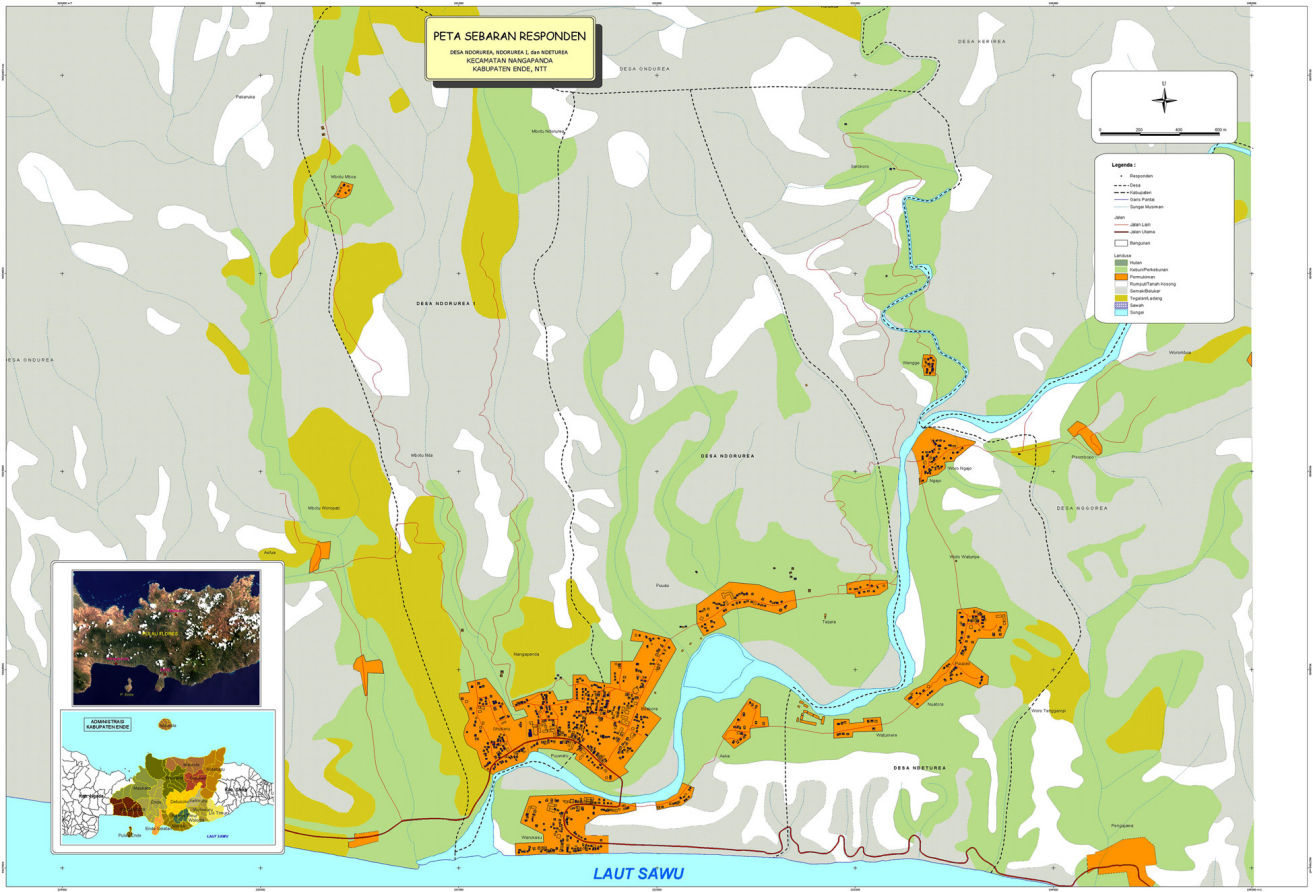
Location of three sub villages enrolled in the study where each black dot represents the house hold.

Nangapanda village, Ende district, Flores Island, Indonesia (Figure 1) is a semi-urban village with an estimated population of 22,000 living in 18 sub villages. It is situated near the Equator, characterized by a high uniform temperature in range of 23 to 33.5°C and humidity is 86 to 95%. Average yearly rainfall is 1.822 mm with around 82 rainy days, especially during November to April and the highest in December until March. The local *Puskesmas* (primary health centre) is responsible for providing health services to people living in the 18 sub villages. Based on the results of a preliminary survey in 2005–2006, three sub villages: Ndeturea (sub village 1), Ndorurea 1 (sub village 2) and Ndorurea (sub village 3) have a high prevalence of malaria cases as reported by the *Puskesmas*. These three sub villages were enrolled in this study. The purpose of the study was explained and informed consent was requested. Informed consent for children was obtained from their parents or guardians. The name, sex, age, and home address of each participant was recorded by the research team.

### Blood collection

Venous blood samples were collected during dry season between June and August 2008 from participants above four years of age in Ndeturea, Ndorurea 1 and Ndorurea. Blood samples (6 ml) were collected in tubes containing sodium heparin as anticoagulant. Eight microlitres and two microlitres from each sample was used to prepare a thick and thin blood smear, respectively; 200 μl was stored at −20°C for real-time PCR and the remainder was used to test a range of immunological markers as part of a larger study [15,17]. The samples were transported to the central laboratory in Jakarta and stored at −80°C.

### Blood parameters and temperature measurement

Haemoglobin level was measured using a blood analyzer system (COULTER® Ac-T diff2™, Beckman Coulter, USA). Haemoglobin levels were divided into normal and anaemic according to criteria used by the Indonesian General Hospital, Cipto Mangunkusumo. The normal values for children (one month to six years), girls (seven to 13 years), boys (seven to 13 years), women (>13 years) and men (>13 years) are haemoglobin 11.5-15.5 g/dl, 12–16 g/dl, 13–16 g/dl, 12–14 g/dl, and 13–16 g/dl, respectively. Cases with haemoglobin levels below these normal values were categorized as anaemic.

The ABO blood typing was performed using monoclonal grouping kit (Fortress Diagnostics Ltd, UK).

The oral temperature was measured using a digital thermometer (General Care, China) and categorized in three groups: low, normal, and high (fever) for temperature level: <36.1°C, 36.1-37.5°C, and >37.5°C, respectively [18].

### Microscopic examination

Duplo slides of both thin and thick smears for malaria diagnosis were made on the same day of blood collection and Giemsa-stained within 48 hours. The guidelines of the WHO Secretariat for the Coordination of Malaria Training in Asia and the Pacific were used as standard operating procedure. The slides were stored in slide boxes at room temperature. Thin and thick smears were examined using 1,000× oil immersion light microscopy for the presence of malaria parasites [19,20]. Microscopic examination of the slides was performed blinded by experienced technicians and approximately 10% of the slides were cross-checked randomly for quality control by a second experienced microscopist at the Department of Parasitology, Medical Faculty, University of Indonesia.

### DNA extraction

Parasite DNA was isolated from 200 μl blood using QIAamp DNA-easy 96-well plates according to the manufacturer’s recommendations (Qiagen, Hilden, Germany). In each sample, 10^3^ PFU/ml phocin herpes virus-1 (PhHV-1) was added within the isolation lysis buffer to serve as an internal control [21]. DNA was stored at 4°C.

### Real-time PCR

*Plasmodium*-specific primers and *P. falciparum*, *Plasmodium vivax*, *Plasmodium ovale* and *Plasmodium malariae*-specific probes were used as described previously [15]. Amplification reactions were performed in white PCR plates, in a volume of 25 μl with PCR buffer (HotstarTaq mastermix; Qiagen, Valencia, USA), a total of 5 mmol/l MgCl2, 12.5 pmol of each *Plasmodium*-specific primer and 15 pmol of each PhHV-1-specific primer, 1.25 pmol of each *P*. *falciparum*, *P*. *vivax*, *P*. *malariae*-specific XS-probes, and PhHV-1-specific Cy5 double-labelled detection probe, 2.5 pmol of each *P*. *ovale*-specific XS-probes (Biolegio, Nijmegen, The Netherlands) and 5 μl of DNA sample. Amplification comprised of 15 min at 95°C, followed by 50 cycles of 15 sec at 95°C, 30 sec at 60°C and 30 sec at 72°C. Negative and positive control samples for each four species were included in each PCR run.

Amplification, detection, and analysis were performed using the CFX real-time detection system (Bio-Rad Laboratories, USA). The PCR output from this system consists of a cycle-threshold (Ct) value, representing the amplification cycle in which the level of fluorescent signal exceeds the background fluorescence reflecting the parasite-specific DNA load in the sample tested. The amplification is considered to be hampered by inhibitory factors if the expected cycle threshold (Ct) value in the PhHV-specific PCR was increased by more than 3.3 cycles. Positive Ct- values were grouped into three groups: Ct < 30.0, 30.0 < Ct < 35.0 and >35.0 representing a high, moderate and low DNA load, respectively.

DNA isolation and set up of the PCR reactions were performed using a custom-made Hamilton robot platform.

### Data analysis

All data were recorded on standardized case report forms using Excel spreadsheets, stored in an Access database (Microsoft, Redmond, USA) and exported for analysis in SPSS 17.0. Analysis for real-time PCR results was done using CFX Manager™ Software Version 1.0, Bio-Rad. Chi-square distribution for the risk of being infected with a *Plasmodium* species, age, residence, gender and sub microscopic infection were calculated as a proportion of parasite infections detected in these groups. Continuous variables were described by the range and median of all PCR-positive cases and were compared between groups by the Mann Whitney-Non Parametric Test. Statistical significance was considered at p-value <0.05.

## Results

### Study population

**Figure 2:**
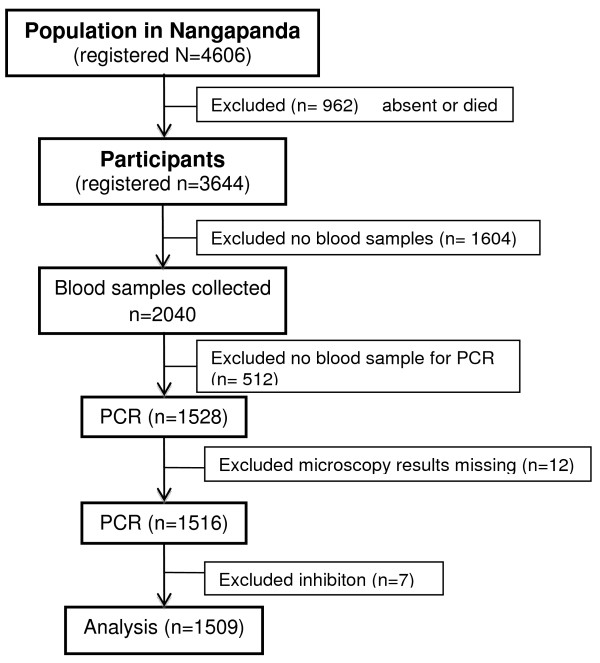
**Flow chart: Cases available for describing the epidemiology of *Plasmodium *infections in Flores Island, Indonesia using real-time PCR.**

A total of 1,516 blood samples were available for microscopy and PCR. Seven samples were excluded due to failure of the DNA isolation or inhibition of the DNA amplification reaction. Figure 2 shows the flow chart of the samples in the study. The final number of samples included in the analysis was 1,509 with ages ranging from four to 79 years, mean and median age of 29 and 27 years, respectively. Participants older than 20 years were over-represented in the analysis group compared to the total population (57.9% *vs* 42.1%). The proportion of males in the analysis group (41.7%) was slightly less than the proportion of males in population (45.3%). A detailed comparison of the population and participants’ characteristics is given in an additional table [see Additional file 1]. Participant characteristics per sub village are described in Additional file 2.

### Microscopy *versus* real-time PCR

The results of microscopy and real-time PCR are summarized in Table 1. Fifty-two (3.4%) of all available samples in the study were positive by microscopic examination and confirmed with real-time PCR. *Plasmodium falciparum*, *P*. *vivax* and *P*. *malariae*-specific DNA amplification was shown in a total of 399 (26.4%) subjects.

**Table 1:** **Number of *Plasmodium *infections detected with microscopy and real-time PCR**

**Species**	Microscopy*	**Real-time PCR**			
	**Total**	**Total**	**Ndeturea**	**Ndorurea 1**	**Ndorurea**
*P. falciparum*	36	172	51	52	69
*P. vivax*	10	158	29	63	66
*P. malariae*	5	22	11	3	8
Mixed Pf and Pv	1	41	3	14	24
Mixed Pf and Pm	0	6	5	1	0
Negative	1457	1110	221	401	488
**Total**	1509	1509	320	534	655
**Total Prevalence**	3.4%	26.4%	30.9%^#^	24.9%^#^	25.5%^#^

*confirmed by real-time PCR.

^#^not significantly different (p = 0.118).

Discrepancies between positive results in the microscopy and real-time PCR were observed in 14 cases. In seven cases real-time PCR revealed another *Plasmodium* species than the species that was determined with microscopy. In another seven samples *Plasmodium*-specific DNA could not be detected, neither with the *Plasmodium* species-specific probes nor when checked for *Plasmodium* genus-specific amplification using agarose gel-electrophoreses.

*Plasmodium falciparum*, *P. vivax* and *P. malariae* parasites were detected with PCR only (i e, were sub microscopic) in 83.1%, 94.5% and 82.1% of the subjects, respectively. Median Ct-values of *P. falciparum*, *P. vivax* and *P. malariae*-specific amplification in sub microscopic cases were higher (i e, lower DNA load) compared to the median Ct-values of microscopy positive subjects 35.3, 36.3 and 36.4 *versus* 30.4, 28.7 and 30.8, respectively (p values ≤0.02).

### Epidemiology of *Plasmodium* infections using real-time PCR

*Plasmodium falciparum*, *P*. *vivax* and *P*. *malariae*-specific DNA amplification was shown in 14.5%, 13.2% and 1.9% of the samples, respectively. The distribution of *Plasmodium* species and infections in each sub village is summarized in Table 1. In all three sub-villages, *P. falciparum*, *P*. *vivax* and *P*. *malariae,* and subjects with mixed infections were detected. Mixed infections of *P*. *falciparum* and *P*. *vivax* were detected in 41 subjects and in six subjects a mixed infection of *P*. *falciparum* and *P*. *malariae* was found. The highest prevalence of *Plasmodium* infections was detected in Ndeturea (30.9%) compared by Ndoturea 1 (24.9%), and Ndorurea (25.5%), but the difference was not statistically significant.

**Figure 3:**
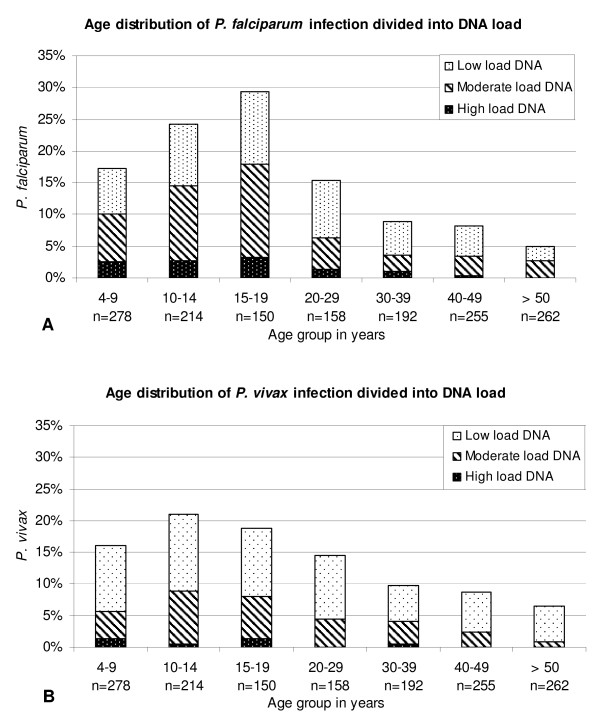
**Age distribution detected with real-time PCR divided in to cases with a high, moderate, and low DNA load of: A. *P. falciparum *infections; B. *P. vivax *infections.**

Figure 3 shows the parasite-specific DNA loads of *P*. *falciparum* and *P*. *vivax* in the different age groups. A high load of *Plasmodium* species-specific DNA (Ct <30) was found for *P. falciparum, P*. *vivax* and *P*. *malariae* in 10.5%, 4% and 14.3% of the positive cases, respectively. A moderate DNA load (Ct 30–35) was found for *P*. *falciparum* (43.8%), *P*. *vivax* (31%), and *P*. *malariae* (39.3%) and a low DNA load (Ct > 35) was detected for *P*. *falciparum* (45.7%), *P*. *vivax* (65%) and *P*. *malariae* (46.4%). *Plasmodium malariae* was not detected in subjects below five years of age.

### Factors associated with *Plasmodium* infection

Table 2 summarizes the *Plasmodium* species-specific real-time PCR results in correlation with age, gender, haematological parameters, and body temperature. The prevalence of *P. falciparum* in subjects younger than 20 years (22.4%) was significantly higher compared to the prevalence found in subjects older than 20 years (8.7%) (p ≤ 0.001). The same was found for the prevalence of *P. vivax* in the young and older age group, 18.4% and 9.3%, respectively (p ≤ 0.001). The median Ct-values of the *P. falciparum* PCR in subjects below 20 years were lower compared to subjects of 20 years and older, 34.2 and 36.0 respectively (p = 0.003). The median Ct-values in the *P. vivax* PCR were not significantly different in the young and older age group. Both prevalence and median Ct-values of the *P. malariae* PCR in subjects below 20 years were not significantly different compared to the subjects older than 20 years.

**Table 2:** ***Plasmodium *species-specific real-time PCR results in correlation with age, gender, body temperature, haemoglobin levels, and ABO blood group typing results**

**Variables**			**n total**	**% Positive**	**p-value**^**1**^	**Ct-value median**	**Ct-value range**	**p-value**^**2**^
**Age group** (years)	Pf	< 20	642	22.4	**<0.001**	34.2	24.2-48.7	**0.003**
		≥ 20	867	8.7		36.0	28.3-43.5	
	Pv	< 20	642	18.4	**<0.001**	35.7	26.5-48.3	n.s.
		≥ 20	867	9.3		36.5	28.3-46.1	
	Pm	< 20	642	2.0	n.s.	33.0	27.2-43.7	n.s.
		≥ 20	867	1.7		36.0	29.8-41.0	
Gender	Pf	Male	629	15.9	n.s.	34.0	24.2-46.0	n.s.
		Female	880	13.5		34.9	27.2-48.7	
	Pv	Male	629	15.7	**0.013**	36.0	28.3-43.2	n.s.
		Female	880	11.4		36.1	26.5-48.3	
	Pm	Male	629	1.9	n.s.	36.6	28.8-43.7	n.s.
		Female	880	1.8		32.1	27.2-42.7	
**Haemoglobin**	Pf	Normal	400	12.8	**<0.001**	34.9	27.1-43.5	**0.02**
		Anaemia	138	30.4		32.7	25.1-42.0	
	Pv	Normal	400	12.5	n.s.	36.9	28.3-48.3	**0.01**
		Anaemia	138	15.9		34.2	26.5-41.6	
	Pm	Normal	400	2.0	n.s.	36.3	28.8-38.1	n.s.
		Anaemia	138	2.9		32.1	30.7-40.7	
Blood Group	Pf	A	320	12.5		35.3	25.1-42.6	
		B	315	13.0		34.1	25.9-43.4	
		O	564	13.1	n.s.^#^	34.3	24.2-78.7	n.s.^#^
		AB	64	18.8		37.2	29.3-38.8	
	Pv	A	320	10.3		36.2	28.3-46.1	
		B	315	15.9		36.3	31.1-43.2	
		O	564	12.4	n.s.^#^	36.3	27.7-44.2	n.s.^#^
		AB	64	20.3		35.8	28.7-39.0	
	Pm	A	320	1.9		35.1	27.2-41.0	
		B	315	1.6		36.0	28.0-36.7	
		O	564	2.0	n.s.^#^	34.8	30.7-43.7	n.s.^#^
		AB	64	1.6		0	40.7	
	Pf	<36.5	394	12.4		34.3	24.2-48.7	
		36.5-37.5	1094	14.9		34.5	25.1-46.0	n.s.
		>37.5	8	50.0		34.1	31.5-39.4	
	Pv	<36.5	394	14.2		36.2	28.3-48.3	
Temperature		36.5-37.5	1094	12.7		36.0	26.5-46.1	n.s.
(°C)		>37.5	8	12.5		40.5	40.5	
	Pm	<36.5	394	2.0		31.4	27.9-36.8	
		36.5-37.5	1094	1.8		35.4	27.2-43.7	n.s.
		>37.5	8	0.0		--	--	

^1^ Pearson Chi square test.

^2^ Non Parametric Test (NPT) with Mann Whitney.

^#^ p-value of blood group AB compared to non AB blood group.

n.s. Not Significant.

The prevalence of *Plasmodium* infections in males compared to females was significantly different for *P*. *vivax* 15.7% *versus* 11.4%, respectively (p = 0.01). There was no difference in prevalence of *P*. *falciparum* and *P*. *malariae*. The median Ct-values of *P. falciparum*, *P. vivax* and *P. malariae*-specific amplification in males were not significantly different compared to those in females.

The haemoglobin concentration was measured in 538 of 1,509 of the participants. Normal haemoglobin level was found in 74.3% of the subjects and 25.7% were categorized as anaemic. The number of *P. falciparum-*positive subjects was significantly higher (p ≤ 0.001) in anaemic participants as compared to the subjects with a normal haemoglobin level. There was no difference in the number of *P. vivax* and *P. malariae* positive subjects in the anaemic and non-anaemic group. The median Ct-values of *P. falciparum* and *P. vivax* -specific amplification were lower in the anaemic cases compared to the non-anaemic subjects (p = 0.02 and p = 0.01 respectively).

Blood grouping was performed in 1,263 of 1,509 subjects. Blood group A, B, AB, and O was found in 25.3%, 24.9%, 5.1% and 44.7% of the subjects, respectively. The highest prevalence of *Plasmodium* infections was found in subjects with blood group AB as compared to the other blood groups. The prevalence and median Ct-values of all three *Plasmodium* species separately were not different between the subjects with different blood groups.

Body temperature was recorded in 1,496 of 1,509 participants. A low body temperature (<36.1°C) was found in 26.3% of the participants, a normal body temperature (36.1-37.5°C) in 73.1% and eight of 1,509 (0.5%) showed a high body temperature (>37.5°C). There was no difference in the number of *Plasmodium* infections in the cases with a low and normal body temperature. *Plasmodium falciparum* was detected in four of eight subjects with a high body temperature. However, the number of subjects in this group was too small for statistical analysis.

## Discussion

In the present population-based study on Flores Island, Indonesia, *P. falciparum, P. vivax, P. malariae* and mixed infections were detected with PCR in 11.4%, 10.5%, 1.5%, and 3.1% of the participants, respectively. Only 87% of all infections were revealed using real-time PCR. The higher sensitivity of the real-time PCR is reflected in the lower DNA load in microscopy-negative subjects compared to the DNA load found in microscopy-positive subjects.

*Plasmodium falciparum* prevalence increased up to 15–19 years of age and decreased in the older age groups; also the parasite load was significantly higher in the participants under 20 years whereas the prevalence of *P. vivax* started to decrease somewhat earlier in age. In areas with higher transmission intensity, such as Irian Jaya and in Africa, the peak in prevalence and parasitaemia is usually observed at a younger age due to an earlier acquired immunity [22-24].

Although only significant for *P. vivax,* the numbers of malaria infections were found higher in males than females. This could be more due to working outdoors in the field resulting in a higher exposure to mosquito bites, but then higher *P*. *falciparum* and *P*. *malariae* infections would be expected. This was also shown in a study performed in India [25] but in contrast with a study in Popondeta, Papua New Guinea, located close to the eastern part of Indonesia, where no significant difference was found in malaria prevalence between males and females [26]. A more detailed statistical analysis of links between gender, work, local ecological, environmental, economic and cultural factors, which might influence exposure to risk of infection and the disease, has been planned for the future.

Clearly anaemia is a well-known phenomenon associated with malaria disease, but also in the present study among apparently healthy individuals, anaemia was strongly correlated with the prevalence and load of *P. falciparum* infections, which is in agreement with other studies in asymptomatic subjects [27-32]. In the present study, anaemia was also correlated with the load of *P. vivax* infections. Although anaemia is a known complication in cases with severe *P. vivax* malaria, it is less known in latent *P. vivax* cases and might be underestimated due to misdiagnosis. Obviously anaemia is a multifactorial clinical phenomenon [33] and no correction was performed for potential other causes.

Several studies were performed to understand the contribution of ABO blood group antigens in protection against *Plasmodium* infections. Although meta-analysis has shown that blood group O provides selective advantages against severe malaria, other studies were unable to link ABO blood groups to the incidence of malaria [34-37]. In the present cross-sectional study, the prevalence of *P. falciparum* and *P. vivax* was higher, although not significantly in subjects with blood group AB compared to subjects with the other blood groups. This seems in contrast with previous findings; however, in the present study mostly sub microscopic *Plasmodium* infections were found using real-time PCR whereas previous studies used microscopy to detect malaria parasites. Further studies in areas of different endemicity using *Plasmodium* PCR will be needed to elucidate the role of ABO blood groups in acquiring infection and the outcome of disease in malaria.

The real-time PCR procedure as described in this paper is not suitable to use as a point of-care (POC) test in “treatment on diagnosis” control strategies. The high-throughput, however makes it very appealing to transport samples to a central laboratory for monitoring the effect of mass drug administration programs [38]. Moreover, nucleic acid based POC assays are in development as well, such as LAMP (loop-mediated isothermal amplification), nucleic acid amplification protocols directly from blood without DNA extraction, and lateral flow detection of PCR products [39-42].

In the present study real-time PCR has proved to be a powerful diagnostic tool providing novel insights into the epidemiology of malaria infections in a low transmission area. The high number of sub microscopic infections have important implications in the transmission dynamics [43-48] and have to be considered in novel elimination strategies [38,49-51]. Moreover, these low density malaria infections appear to play a role in eliciting or maintaining humoral immune responses [48,52,53] and therefore should be taken into account in, for example studies on the immunology in malaria infections and the association with the regulatory responses that may be caused by chronic worm infections interacting with inflammation [15].

## Conclusions

Real-time PCR revealed 7.8 times more *Plasmodium* infection compared to microscopy. Sub microscopic infections are common in this area, over 22% of the subjects in the study show sub microscopic *Plasmodium* infections, which might play an important role in transmission. Real-time PCR provides more insight into the epidemiology of *Plasmodium* infections, which could be applied for preventing, monitoring, and evaluation of novel programs for the elimination of malaria.

## Competing interests

The authors declare that they have no competing interests.

## Authors’ contributions

MY developed the study and is the Dutch coordinator of the ImmunoSPIN program. TS developed the study and is the Indonesian coordinator of the ImmunoSPIN program. ES contributed to the study coordination and advised on data collection. AEW, FH, and LJW contributed to setting up the field study, recruitment, and follow up and data collection. AJFL contributed to advice on manuscript. LvL and JJV led the work on real-time PCR detection of parasites. MMMK contributed to fieldwork, data collection, performed real-time PCR work. JJV and EATB helped to interpret the results of molecular diagnostic. MMMK and JJV contributed to the statistical analysis of the results and drafted manuscript. All authors read and approved the final manuscript.

## Supplementary Material

Additional file 1 Comparison of age, gender, and residential area of the total population and participants. Click here for file

Additional file 2 Age distribution and gender of participants from three sub villages in Nangapanda, Ende district. Click here for file
