# Supplementary material for: Epidemiology of Plasmodium infections in Flores Island, Indonesia using real-time PCR
Source: Malar J. 2013 May 24;12:169. doi: 10.1186/1475-2875-12-169 (PMC3679745; doi:10.1186/1475-2875-12-169)
Supplement: Additional file 1 — Comparison of age, gender, and residential area of the total population and participants. [file 1475-2875-12-169-S1.pdf]

Additional file 1 Comparison of age, gender, and residential area of the total population and participants.

Characteristics		Population (%) N= 3644	Analysis group (%) N=1509	P value
Age range (median) in years		0-93 (22)	4-79 (27)	
Age (years)	4-9	20.5	16.6	0.001
	10-14	13.4	14.7	0.221
	15-19	13.1	10.7	0.021
	20-29	12.8	10.4	0.018
	30-39	13.0	12.7	0.823
	40-49	12.8	16.6	0.000
	50 >	14.5	18.2	0.001
	Total	100	100	
Age (years)	Young (< 20)	47.0	42.1	0.001
	Adult (≥ 20)	53.0	57.9	
	Total	100	100	
Gender	Male	45.3	41.7	0.019
	Female	54.7	58.3	
	Total	100	100	
Villages	Ndeturea	18.7	21.2	0.038
	Ndorurea 1	33.2	35.4	0.122
	Ndorurea	48.2	43.4	0.002
	Total	100	100	
