# Supplementary material for: Epidemiology of Plasmodium infections in Flores Island, Indonesia using real-time PCR
Source: Malar J. 2013 May 24;12:169. doi: 10.1186/1475-2875-12-169 (PMC3679745; doi:10.1186/1475-2875-12-169)
Supplement: Additional file 2 — Age distribution and gender of participants from three sub villages in Nangapanda, Ende district. [file 1475-2875-12-169-S2.pdf]

Additional file 2 Age distribution and gender of participants from three sub villages in Nangapanda, Ende district

**Age Group**	**Total** n total (% male)	**Sub Villages** n total (% male)		
		Ndeturea	Ndorurea 1	Ndorurea
4-9	278 (52.52)	66 (59.10)	88 (54.55)	124 (47.58)
10-14	214 (50.00)	36 (58.33)	78 (43.59)	100 (52.00)
15-19	150 (42.00)	22 (22.73)	64 (51.56)	64 (39.06)
20-29	158 (22.15)	39 (12.82)	57 (28.07)	62 (22.58)
30-39	192 (27.60)	45 (22.22)	71 (26.76)	76 (31.58)
40-49	255 (41.96)	48 (33.33)	89 (44.94)	118 (43.22)
50<	262 (45.04)	64 (40.63)	87 (48.28)	111 (45.05)
**Total**	**1509 (41.68)**	**320 (38.13)**	**534 (43.45)**	**655 (41.98)**
